# Chemistry and Some Biological Potential of Bismuth and Antimony Dithiocarbamate Complexes

**DOI:** 10.3390/molecules25020305

**Published:** 2020-01-12

**Authors:** Jerry O. Adeyemi, Damian C. Onwudiwe

**Affiliations:** 1 Material Science Innovation and Modelling (MaSIM) Research Focus Area, Faculty of Natural and Agricultural Science, North-West University, Mafikeng Campus, Private Bag X2046, Mmabatho 2735, South Africa; jerryadeyemi1st@gmail.com; 2 Department of Chemistry, Faculty of Natural and Agricultural Science, North-West University, Mafikeng Campus, Private Bag X2046, Mmabatho 2735, South Africa

**Keywords:** bismuth, antimony, dithiocarbamate, biological activities, structural properties

## Abstract

Interest in the synthesis of Bi(III) and Sb(III) dithiocarbamate complexes is on the rise, and this has been attributed to their wide structural diversity and their interesting application as biological agents and in solid state/materials chemistry. The readily available binding sites of the two sulphur atoms within the dithiocarbamate moiety in the complexes confers a wide variety of geometry and interactions that often leads to supramolecular assemblies. Although none of the bismuth or antimony metals are known to play any natural biological function, their dithiocarbamate complexes, however, have proven very useful as antibacterial, antileishmanial, anticancer, and antifungal agents. The dithiocarbamate ligands modulate the associated toxicity of the metals, especially antimony, since bismuth is known to be benign, allowing the metal ion to get to the targeted sites; hence, making it less available for side and other damaging reactions. This review presents a concise chemistry and some known biological potentials of their trivalent dithiocarbamate complexes.

## 1. Introduction

The sustained interest in the dithiocarbamate complexes of bismuth and antimony [1] could be attributed to several reasons. These include their structural diversity, which range from monomeric to polymeric supermolecular assemblies, their unique application as biological agents/catalysts, and utilisation in materials and surface chemistry [1,2,3,4]. In addition, the straightforwardness of their synthesis has also contributed to their increased attention [4]. The two elements, bismuth (Bi) and antimony (Sb), are both in Group 15 (pnictogen) of the Periodic Table [5,6]. Bismuth is the heaviest element of this group and is often referred to as a “green” metal due to its benign nature and low toxicity compared to the other group 15 elements such as arsenic and antimony [5]. It has been known since ancient times, and has often been confused with tin and lead due to its position (diagonally and horizontally) in the Periodic Table, which accounts for the similar properties that bismuth shares with these other metals [7,8]. However, its low toxicity remains the major difference in properties when compared to these metals. In fact, most bismuth compounds have a toxicity much lower than sodium chloride [7]. Studies have shown that their low toxicity emanates from their insolubility in most neutral aqueous solutions such as biological fluids [7]. In some organic syntheses, Bi compounds have been used as a Lewis acids in arylation, cycloaddition, alkylation, aldolisation, epoxide ring opening, esterification, hydroalkylation oxidation, and hydroarylation reactions [9].

The trihalide derivatives of the pnictogen group are found to behave as weak acceptors toward other ligands, and these Lewis acidic properties increase down the group for a given halide [10]. The high abundance and low cost of the Bi(III) trihalide derivatives, make them an ideal starting material for the synthesis of transition metal–main group complexes. This is possible because the size of the bismuth centre allows for three and higher coordination through the linking of one or more transition metal fragments. Thus, the possibility of variation in the metal fragments confers great access to a wide range of available structures with metal carbonyl, cyclopentadienylcarbonyl, and sandwich structures of bismuth already in existence [11]. Furthermore, immense progress has been made in the organometallic chemistry of Group 15 elements [11]. The organometallic derivatives of Sb and Bi are often referred to as stibines and bismuthines, respectively, and they have been widely studied for arsine and phosphine ligands. Stibines (SbR_3_) and bismuthines (BiR_3_) are the heavier analogues of these ligands, and they confer more interesting features that are often less shown in simpler analogues [10]. 

Generally, organometallic complexes show unique physio-chemical properties and have been extensively used in catalysis and as drug candidates [12]. In recent times, due to the useful characteristics of these group of compounds such as structural diversity, possibilities for ligand exchange, redox and catalytic properties, they have been used for diverse medicinal purposes [12,13,14]. This has consequently resulted in the synthesis of an array of medicinally useful compounds, with few already in the clinical trial stages [12,13]. The ligand exchange potential of organometallic complexes, in a similar fashion to ‘ordinary’ metal complexes, is responsible for their wide usage for medicinal purposes. Instead of exchanging a “non-useful ligand” (such as a chloride ion) to form a useful metal adduct, which interrupts the proper function of a precise biomolecules, the released ligand molecule could actually cause a medicinal effect of its own in the biological system [12]. Dithiocarbamate is one such useful ligand capable of conferring a useful medicinal effect in a biological system due to its proven capacity to inhibit enzymes and ultimately affect biological environments [14]. 

Dithiocarbamates are the amides of dithiocarbamic acid and are capable of forming stable metal complexes due to their outstanding coordination properties [14]. They have a wide range of applications and chemistry, consequently resulting in their versatility. Due to the insoluble nature of dithiocarbamate and its compounds, they have been widely used in inorganic analysis and also for the separation of different metal ions in high-performance liquid chromatography (HPLC) [5,6,7,8,9,10] and capillary gas chromatography (GC) [15]. Additionally, they have found usage as pesticides, fungicides, and as accelerators in rubber vulcanisation [15]. The readily available strong binding site of the two sulphur atoms within their structure confers a large ionic radii; hence, dithiocarbamates have shown the ability to bind strongly in nine different modes to a metal atom [16,17,18]. This functional group [=CS_2_] of the dithiocarbamate family is often obtained through a CS_2_ insertion reaction with either a primary or secondary amine compound [18]. A resonance between these two sulphur atoms consequently allows various binding modes with different metals [18]. Several studies, reactivity and the synthesis of different transition metal dithiocarbamate compounds, with diverse structural geometry have been carried out [19]. This has been due to their ease of synthesis [19] and their diverse applications in medicine, organic synthesis, agriculture, and recently as precursors for metal sulphide nanoparticles [18]. 

Bismuth and antimony compounds have been reported to show great potential as medicinal agents [5,6] but their complexes with dithiocarbamate have not been extensively studied. However, the few studied ones have shown a wide range of chemistry and biological activities. It is against these backdrops that we review some of the recent studies on the chemistry and the biological potential of different derivatives of Bi and Sb dithiocarbamate complexes including their respective organometallic dithiocarbamate complexes. 

## 2. Chemistry of Bismuth and Antimony Dithiocarbamate Complexes 

The etymology of the name “bismuth” is thought to originate from the German word “Wismuth”, meaning white mass and exists primarily as bismite (bismuth oxide) and bismuthinite (bismuth sulphide) ores. It is usually found as a by-product in the mining of Cu, Pb, and Sn [20]. It is the rarest element and the least abundant of the pnictogens or Group 15 elements; is monoisotopic in nature and is often considered the heaviest stable element with a half-life of 1.9 × 109 years [20]. Bismuth has both semi-metallic and metallic properties as opposed to antimony, which is considered to be a metalloid. It has a ground state electronic configuration of [Xe]4f^14^5d^10^6s^2^6p^3^ and occurs in two oxidation states of +3 and +5. In most cases, the 6p electrons are used for bonding, mostly in a +3 oxidation state to form a compound. Thus, in such cases, the 6s^2^ pair electrons are inert, which could cause a stereo-chemical effect [20]. However, the pair of electrons on the 6_S_ orbital could be involved in bonding, as in the case of organobismuth(V) compounds [21]. The only Bi(V) halide known is BiF_5_. Bi(V) is a strong oxidant and is not stable in a biological system [22]. One prominent example of bismuth compounds is the organobismuth compound, which possesses at least one carbon to bismuth bond within its structure. In organobismuth compounds, bismuth could exist in +3 or a +5 oxidation states. A deeper understanding of this class of compounds is still developing as only few reports are available. Two key factors have been identified to contribute to the coordination chemistry of bismuth in the oxidation state of +3:

i The 6_S_ orbital is stabilised by relativistic effects resulting in less availability of the two 6s^2^ electrons for bonding and a concomitant reduction in Lewis basicity (inert pair effect).
ii Possibility for an extension in coordination around the Bi(III) centres, especially when bonded to an electronegative atom or group. This occurs due to the significant degree of Lewis acidity resulting from the availability of the unoccupied d-orbital and weak shielding of the 4f electrons, referred to as lanthanide contraction [21,22].

Bi(III) is often referred to as the borderline Lewis acid based on the Hard Soft Acid Base (HSAB) theory; hence, has the capacity to form weak complexes [23]. Generally, Bi(III) easily forms complexes with ligands bearing the oxygen, nitrogen, and the sulphur donor atoms, which makes them suitable for biological systems [22]. The ionic radius of Bi(III) offers the possibility for complex formation with a higher coordination number involving a wide variety of geometries [24,25]. It is well known that bismuth has the capacity to interact with biological systems such as nucleotides, nucleosides, amino acids, peptides, and proteins. It is, therefore, easy to understand its biological targets [20]. Many complexes of Bi with different organic moieties have proven to be useful as antimicrobial [4], antileishmanial [26], and anticancer [20] agents. 

Similar to the origin of bismuth, antimony was first referred to in its natural sulphide form as “Stem”; hence, the Greek name “Stimmi” and the Latin “Stibio” [27]. The name “antimony”, however, has long been designated to the natural sulphide form as opposed to what it is been known as in the last two to three centuries, where chemists have adopted this name specifically for its pure metal [27]. Its most important ore, stibnite (Sb_2_S_3_), was used in ancient times as a dark painting material on women in ancient Egypt and Persia [28]. Some natural occurring minerals that contain arsenic have been identified over the years and these include ullmanite (NiSbS), tetrahedrite (Cu_3_SbS_3_), and a number of other complex sulphides [28]. The pure form of antimony is silvery shining white in nature, which when slowly cooled, crystallises in the hexagonal system, isomorphic grey form, and on rapid cooling, becomes granular and crystalline in nature [29]. As a general trend within Group 15 metals, there is a progressive increase in metallic character down the group and most of these elements exist in +3 and +5 oxidation states [28]. Antimony, however, has three valence electrons of +3, +4, and +5 and also bears similar chemical property to arsenic, with a ground state electronic configuration of [Kr] 4d^10^5s^2^5p^3^ [29,30]. It is close to the zinc group as well as to germanium. Antimony bears such a resounding resemblance to germanium that the discoverer of the latter called it the first Eka-antimony of Olendelejeff [29]. It is a metalloid and exists in four allotropes, of which the most stable is the greyish metallic form. This metallic form is one of the unusual metals known to expand upon freezing. It is mostly found in its trivalent and pentavalent states [31]. About thirty isotopes of antimony are known, and the most important was reported by Smith in 1973 where ^121^Sb and ^123^Sb occur naturally [32]. A major difference between the coordination chemistry of antimony and other metals in Group 15 is the great stability shown by its +5 oxidation state, which forms wide range of complexes with a large number of ligands [22,33]. In its +3 oxidation state, antimony is a weak base and possesses an electropositive character [29].

**Figure 1:**
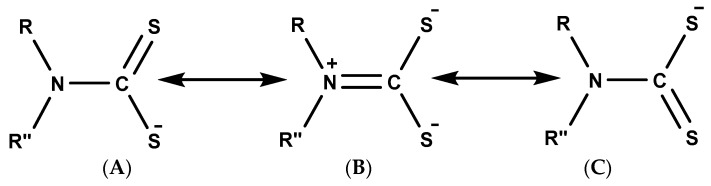
Resonance structure of dithiocarbamate.

In the past decades, attention has been given to antimony and bismuth complexes involving the 1,1-dithiolates ligands due to their wide structural diversity [34]. Their geometry has also been found to be influenced by a lone pair of electrons and the nature of the group attached to the metal centre and/or the dithiolate ligand [34]. Anions of dithiolate/dithiocarbamate ligands have a general formula –S_2_CNR′_2_ and are often known for their good metal-coordination properties [35]. Generally, dithiocarbamates exist in three different resonant forms, as shown in Figure 1 [14]. In the resonant forms A and C, there is a single bond between the carbon and the nitrogen atom, which favours the delocalisation of −1 charge between the two sulphur atoms and the carbon. Thus, both resonance structures favour the stabilisation of soft metals of low oxidation states. The resonance form B is often referred to as the thioureide form [36]. Here, the lone pair electron on the nitrogen is delocalised, resulting in a double bond character between the carbon and nitrogen group. It is in this form that most hard metals of higher oxidation states are stabilised. Hence, the outstanding stability of dithiocarbamate is often due to the contribution of the resonance form B to the overall electronic structure [14,36]. 

**Figure 2:**
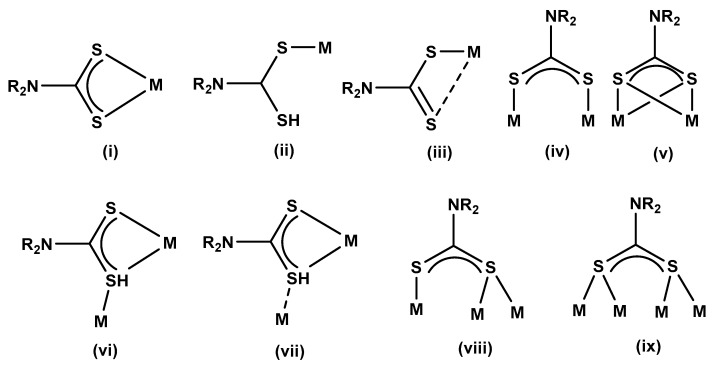
Nine possible coordination modes of dithiocarbamate [15].

Dithiocarbamate ligands are generally prepared by reacting an amine (either primary or secondary) with carbon disulphide, in the presence of a strong base (ammonium hydroxide or alkali hydroxide) to give the corresponding ammonium or alkali salt [37]. The corresponding metal complexes are also often synthesised by the facile reaction of the metal salt with the dithiocarbamate ligand via metathesis [35]. These ligands have the capacity to bind in a variety of ways, as shown in Figure 2, between one to four metals. The commonest mode is the bidentate chelating mode (i) [15]. In this (i) mode, both the sulphur–metal bonds are almost equal and the dithiocarbamate ligands could be considered to possess a net of four electrons [15]. This mode of bonding is common for most transition metals. 

When the bidentate mode of binding is not favoured such as in case (i), due to the demand of other coordinated ligands, the dithiocarbamate binds in a monodentate fashion as in case (ii), as shown in the studies reported by Mahon et al. [38]. This mode of binding has also been found when there is a change in one of the M–S bonds, due to weak coordination of a sulphur atom in the dithiocarbamate moiety [17,35]. In some cases, the sulphur atoms coordinate to the metal in an unequal fashion, resulting in an asymmetric structure (termed anisobidentate) such as in mode (iii) [15]. This type of coordination mode leads to long and short carbon–sulphur bonds, and has been reported for many transition and main group dithiocarbamate complexes [39,40,41,42]. The method of binding in mode (iv) is not particularly common. In this mode of coordination, each of the S atoms separately coordinates to a metal centre [15]. This is mostly found in dithiocarbamate complexes of gold(I) and gold(II) [15]. In the coordination mode (v), the sulphur atoms bind to each metal atom with a bridging coordination between alternating sulphur and metal atoms. Only a few examples are known for this type of binding fashion, and this is mostly found in dirhodium complexes [15]. For the binding mode of (vi) to (ix), the dithiocarbamate ligands bridge with two metal atoms in a number of ways. In mode (vi), all three metal–sulphur interactions have similar bond distances, with the sulphur atoms binding in a symmetrical fashion to the metals. In mode (vii), the interactions are similar, but the third bridging metal sulphur bond is unsymmetrical [15]. In the case of binding modes (viii) and (ix), the dithiocarbamate ligand caps three or four metal atoms, respectively. These coordination modes are mostly restricted to the late transition metals and are very uncommon [15].

The ease of the replacement of hydrogen from the –SH group in dithiocarbamic acid form and their complexes in inorganic analysis is the main reason for their numerous applications [14]. Different methods have been used to synthesise metal dithiocarbamate complexes. However, the most common approach already established for a number of transition metal complexes is the direct ligand addition. This approach most often results in a coordinated anionic ligand and, in a few cases, a second neutral ligand [15]. Different derivatives of antimony and bismuth dithiocarbamate complexes have also been prepared in a similar way.

### 2.1. Synthesis of Antimony and Bismuth Dithiocarbamate Complexes

Different approaches have been used to prepare bismuth(III) and antimony(III) dithiocarbamate derivatives. Although, these metals exist in both +3 and +5 oxidation states, not much literature exists on bismuth(V) dithiocarbamate complexes [1]. Most of the pentavalent dithiocarbamate complexes of the Group 15 metals are dominated by the antimony complexes for both the inorganic and organometallic derivatives. This is because of the redox process and the often facile reaction with ligands bearing the sulphur donor groups [1]. Therefore, the scope of study on the synthesis of both antimony and bismuth, in this current report, is limited to their trivalent inorganic and organometallic dithiocabamate complexes.

The synthesis of antimony and bismuth dithiocarbamate derivatives with a diverse array of structures, have been reported. Fabretti et al. [43] reported an array of metal monomethylsubstituted piperidinodithiocarbamate complexes, which included both antimony(III) and bismuth(III). The characterisation and structure of the compounds, mostly achieved by spectroscopic techniques, indicated that the metals bonded in a bidentate fashion to the piperidinodithiocarbamato anion in a 2:1 and 3:1 ratio, with both Bi(III) and Sb(III) displaying the latter ratio. Other reports made in recent times involved Sb(III) and Bi(III) complexes of dimethyl and diethyldithiocarbamates ligands [42]. These complexes were synthesised by the reaction of SbCl_3_ and BiCl_3_ with either tetramethylthiuram monosulphide, tetramethylthiuram disulphide, or tetraethylthiuram disulphide. The SbCl_3_ salt was reacted with either tetramethylthiuram monosulphide (in methanolic solution) or tetramethylthiuram disulphide (in a mixture of acetonitrile/dichloromethane solutions) to give two polymorphic form of the antimony(III) dimethyldithiocarbamate complex; while tetraethylthiuram disulphide was reacted in a mole ratio 1:1 with BiCl_3_ to give the desired Bi(III) complex. Sb(III) and Bi(III), derived from different secondary amines, with the general formula MCl[S_2_CNR′R″]_2_ (where R′ = benzyl, methyl, sec-butyl, propyl, ethyl, isopropyl, butyl; R″ = cyclohexyl, ethyl, methyl, propyl, isopropyl, benzyl, ethanol), have been reported by Jamaluddin and Baba [44]. These complexes were prepared by the addition of a mixture of CS_2_ in cold ethanol to the respective amine solutions, which was followed with the dropwise addition of a solution of the respective metal chloride salt in cold ethanol to give a pale yellow precipitate of Bi(III) and white Sb(III) complexes. Bismuth(III) complexes of dimethyldithiocarbamate and diethyldithiocarbmate have been reported by Arda et al. [45]. The synthesis of these complexes involved the reaction of bismuth(III) halide (BiX_3_, X = Br or I) with tetramethylthiuram monosulphide, tetramethylthiuram disulphide or tetraethylthiuram disulphide in a 1:1 ligand to metal ratio. In another report, a mixture of small pieces of bismuth metal were complexed with dimethyldithiocarbamate and diethyldithiocarbmate ligands in toluene and refluxed for 3 h. The unreacted traces of both the ligand and the metals used were removed by filtration, and the resultant yellow solutions were reduced to ca. 1/3 of their volume and cooled to room temperature. The obtained solid was then washed in ether and dried in vacuo [46]. The synthesis of these complexes from the metal salts are not only restricted to the halogen salt, their oxides, BiONO_3_ and Sb_2_O_3_, have also been used by Venkatachalam et al. [47]. Their respective complexes derived from *N*-methylaminoethanoldithiocarbamato and *N*-methylaminoethanol dithiocarbamate ligands were reported. Their different structures have been ascertained by various spectroscopic and single crystal analysis techniques. There has been individual reports on either Bi(III) or Sb(III) complexes. Bimuth(III) *N*-ethyl cyclohexyldithiocarbamate complexes have been synthesised in situ [3] by the reaction of *N*-ethyl cyclohexylamine with CS_2_ in the presence of aqueous KOH and in an ice cold condition. After the ligand formation in the solution was ascertained, it was followed by the addition of BiCl_3_ in a 3:1 ratio to afford a yellowish orange precipitates and was recrystallised in chloroform and ethanol solutions.

Additionally, many reports have been made on the Sb(III) counterpart and most of these complexes have been derived from metal halide sources. Yin et al. [48] reported some Sb(III) complexes derived from dithiocarbamate [R_2_NCS_2_ =OC_4_H_8_NCS_2_ (1), C_2_H_5_NC_4_H_8_NCS_2_ (2), Me_2_NCS_2_ (3), C_4_H_8_NCS_2_ (4), C_5_H_10_NCS_2_ (5), Bz_2_NCS_2_ (6), Et_2_NCS_2_ (7), and (HOCH_2_CH_2_)_2_NCS_2_ (8)], which were made from different dialkyl-secondary amines. These complexes were synthesised by the reactions of Sb(III) halides with dithiocarbamate ligands in 1:2 or 1:3 stoichiometries using acetone and tetrahydrofuran. The obtained solids were all recrystallised in ethanol, dichloromethane, chloroform, and acetonitrile (or a mixture of these solvents). In the synthesis of mixed bis(morpholine-4-dithiocarbamato-S,S′) antimony(III) complexes, an intermediate compound of the Sb(III) dithiocarbamate complex was obtained [49]. This complex was synthesised by the reaction of a hexane solution of antimony(III) bromide with a hexane solution of potassium morpholine-4-dithiocarbamate ligand, and the product was obtained after refluxing for 5 h. Another report by Baba et al. [50] involved the reaction of antimony(III) trichloride and *Tris*(*N*-cyclohexyl-*N*-methyl dithiocarbamato-S) ligands in ethanol (1/3 molar stoichiometry) [50]. The obtained precipitate was further recrystallised in ethanol to afford the desired metal complex.

**Figure 3:**
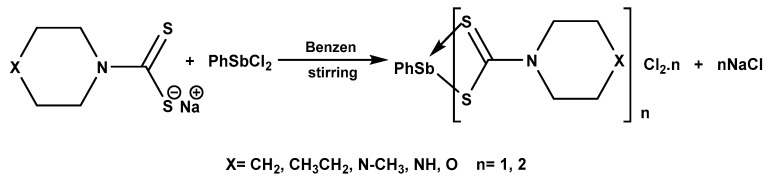
Synthesis of phenylantimony(III) dithiocarbamate complexes.

Organobismuth(III) and oraganoantimony(III) dithiocarbamate complexes have also been studied. Sharma et al. [51] reported a new class of phenylantimony(III) dithiocarbamate complexes (Figure 3). Different derivatives of the sodium salt of cyclic dithiocarbamates in 1:1 and 1:2 molar ratios gave varieties of complexes which were fully characterised using various spectroscopic techniques.

In general, for dithiocarbamate complexes, properties such as solubility in organic and non-organic solvents and the thermal decomposition pattern of these complexes are influenced by the nature of the dithiocarbamate ligand attached to the metal. The colour of the complexes remains the same, although sometimes appears in different shades of the expected colourations. Thus, bismuth complexes appear yellowish orange while antimony complexes appear as a white solid in their trivalent solid states.

Spectroscopic techniques are vital in the characterisation of dithiocarbamate compounds. The assignment of Fourier-Transform Infrared (FT-IR) spectral bands are fully established in the literature [52,53]. The spectra are often characterised by three main regions, primarily associated with the stretching vibrational bands of υ(C-N), υ(C-S), and υ(M–S) [54,55,56], which are found in the frequency ranges of 1450–1550, 950–1000, and 350–450 cm^−1^, respectively [14]. The shift in the stretching vibrational bands of υ(C-N) by 15 cm^−1^ upon complexation with a metal often suggests a partial double bond character. This band appears in a region between the single bond *v*(C–N) and the double bond *v*(C=N) stretching vibrations [14,57,58]. The appearance of a single or split band in the range 1000–950 cm^−1^ suggests a bidentate or a monodentate coordination mode respectively with the metal centre [59]. The –CS_2_ of the thiouride group, usually found in lower field on the ^13^C spectra around 200 ppm, are known to move upfield upon complexation with a metal [51].

### 2.2. Structure of Inorganic and Organometallic Bi(III) and Sb(III) Dithiocarbamate Complexes

Crystal structures of both inorganic and organometallic Bi(III) and Sb(III) dithiocarbamate complexes have been obtained and studied over the years. These studies have established that dithiocarbamate ligands bond to both the Bi(III) and Sb(III) centre in a number of ways, which are mostly bidentate, monodentate, and anisobidentate in nature. Unlike the oxo-ligand derivatives, the dithio-derivatives have been reported to prefer discrete monomeric structures and, if there are any, a weak secondary interaction that inhibits the formation of a large molecule in a true sense [1]. In the Bi(III) and Sb(III) complexes of the ligand systems with the S^∩^S, such as dithiocarbamate, the M–S bonds are often asymmetrically coordinated, with the difference between the two metal–sulphur bond distances decreasing in the order, Sb > Bi [1]. The stereochemical activity of the *ns*^2^ lone pair [n = 5(Sb), 6(Bi)] causes these elements to adopt wide and varied coordination geometries. Hence, they could adopt a regular octahedral geometry if the lone pair is suppressed by hybridisation with p- and d-orbitals [1]. However, this is rare and the commonest occurrence in the geometry of these metal atoms is the distorted octahedral geometries (particularly for ML_3_ complexes with short M–S bonds trans to long bonds). This has resulted in an array of coordination environments resembling a trigonal pyramid, square pyramid, trigonal bipyramid, or antiprisms. For the organometallic derivatives, the alkyl group attached to the metal centre also plays a crucial role in the final outcome of the geometry [1].

**Figure 4:**
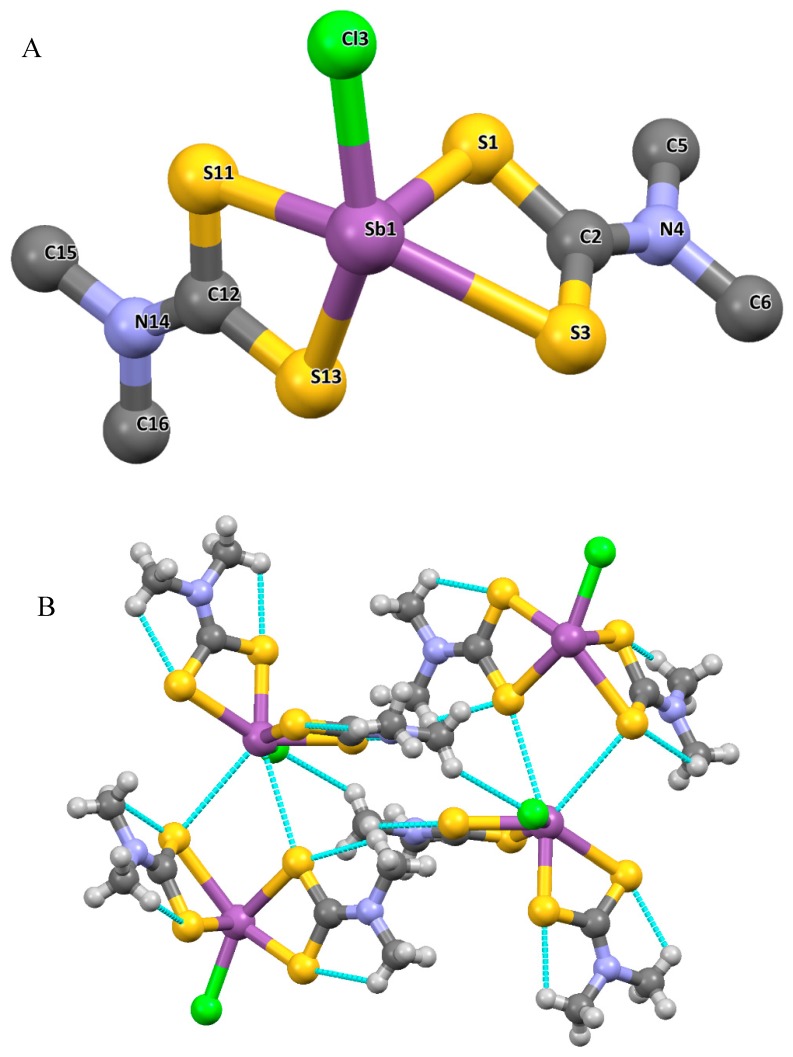

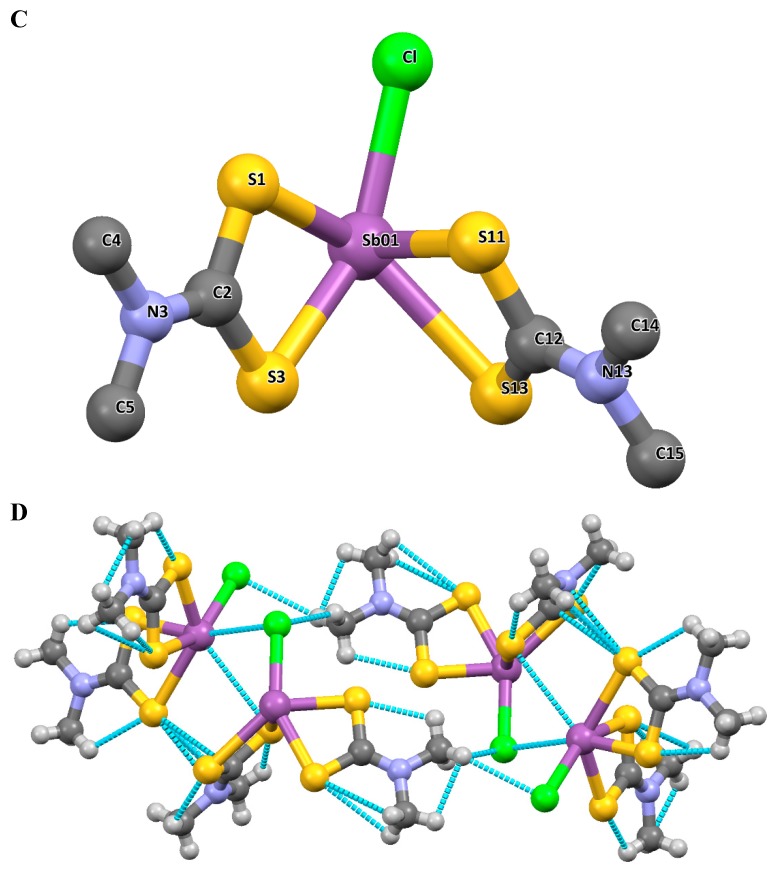

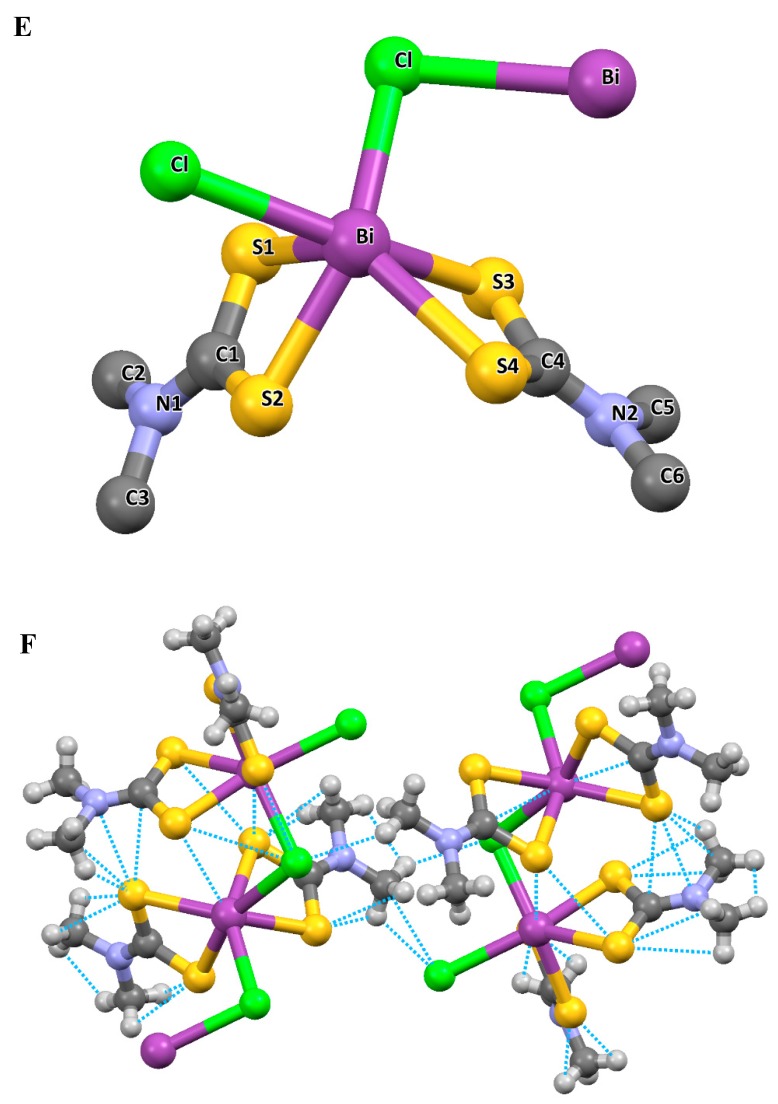

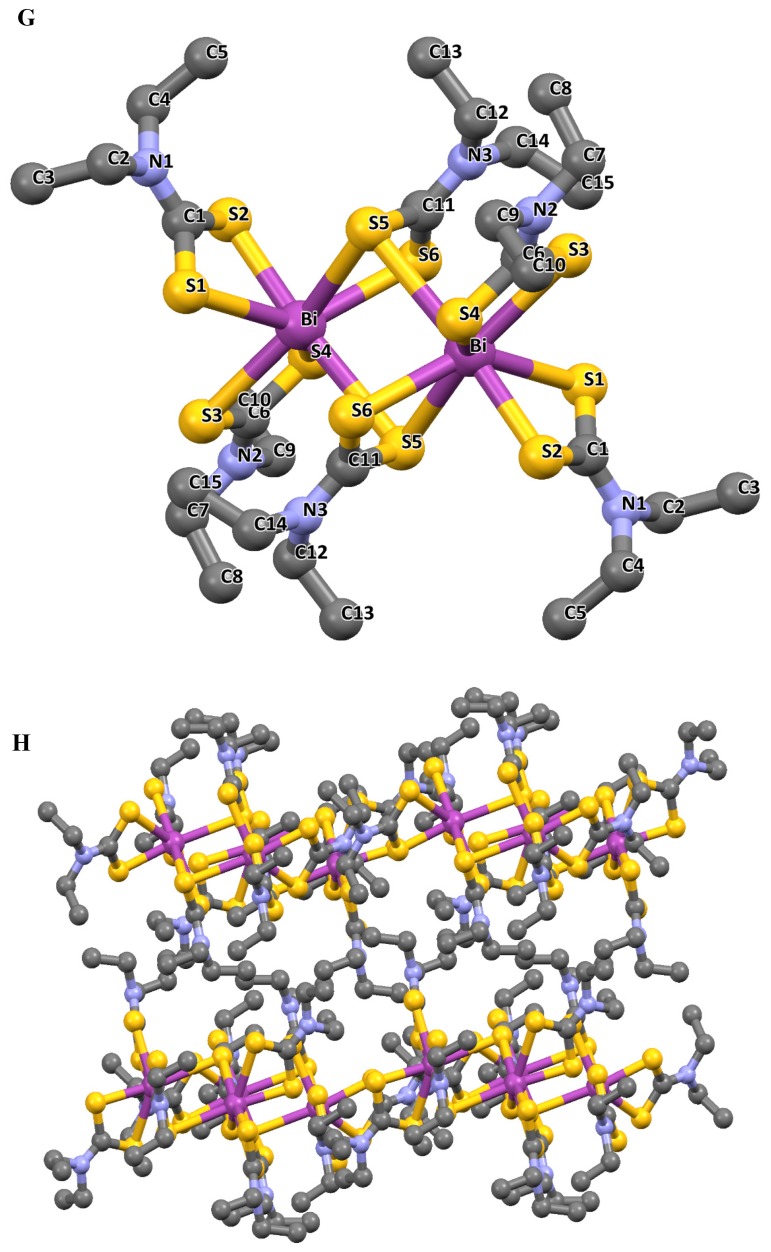
(**A**) Crystal structure and the labelling scheme of [SbCl(Me_2_DTC)_2_]n (1a). (**B**) Intermolecular, μ_2_—S····Sb interactions leading to polymerisation of [SbCl(Me_2_DTC)_2_]n (1a). (**C**) Crystal structure and the labelling scheme of [SbCl(Me_2_DTC)_2_]n (1b). (**D**) Intermolecular μ_2_—S····Sb interactions leading to polymerisation of [SbCl(Me_2_DTC)_2_]n (1b). (**E**) Crystal structure and the labelling scheme of [Bi(Me_2_DTC)_3_]_2_}(2). (**F**) Intermolecular μ_2_—S····Bi and μ_2_—Cl····Bi interactions leading to polymerisation in complex {[Bi(Me_2_DTC)_3_]_2_}(2). (**G**) Crystal structure and the labelling scheme complex {[Bi(Et_2_DTC)_3_]_2_}(3). (**H**) is the crystal packing viewed along the best axis. All images are redrawn from [42], with permission from Elsevier (copyright 2019).

A few examples presented here show the diverse geometry of these complexes. For instance, the structure of Sb(III) and Bi(III) complexes of dimethyldithiocarbamate [Me_2_DTCH], and diethyldithiocarbamate [Et_2_DTCH] have been reported and the structures are presented in Figure 4A–H [42]. Two different polymorphs [SbCl(Me_2_DTC)_2_]n (1a/1b) of the Sb(III) tris(dimethyldithiocarbamate) were obtained, in addition to the structures of Bi(III) tris(dimethyldithiocarbamate) {[Bi(Me_2_DTC)_3_]_2_}(2) and Bi(III) tris(diethyldithiocarbamate) {[Bi(Et_2_DTC)_3_]_2_}(3). In complexes [SbCl(Me_2_DTC)_2_]n (1a/1b) and {[Bi(Me_2_DTC)_3_]_2_}(2), the metal centre was bonded in a five coordinate arrangement, while bearing a pendant Cl^−^ atom. The dithiocarbamate sulphur atoms and the metal centre were coordinated in an anisobidentate fashion (two short and two long M–S bonds lengths) to each other. Although, it might appear as if one of the long Sb–S bonds was uncoordinated to the metal, this was not the case because the distance between the sulphur and the metal atom was well within the expected Sb–S lengths (varying from 2.482 to 3.009 Å) [42]. From Figure 4A–D, it could be observed that the chloride ions from the metal salt were still present in the final structure. This has been attributed to the incomplete substitution of the chloride ion by the ligand as a result of the mole ratio (of the metal to ligand) used in the preparation of these complexes. Complexes [SbCl(Me_2_DTC)_2_]n (1a/1b) and {[Bi(Me_2_DTC)_3_]_2_}(2) gave a polymeric structure with a distorted square pyramidal geometry in each case, while a dimer with distorted octahedral geometry was formed in complex {[Bi(Et_2_DTC)_3_]_2_}(3) regardless of the mole ratio used. According to the report, the polymorphic form of [SbCl(Me_2_DTC)_2_]n (1a/1b) resulted from the different preparative procedure used. In both complexes [SbCl(Me_2_DTC)_2_]n (1a/1b) and {[Bi(Me_2_DTC)_3_]_2_}(2), the dithiocarbamate ligand was anisobidentate μ_2_—bridging. In the polymorphic form [SbCl(Me_2_DTC)_2_]n (1a), one of the bridging interactions, μ_2_—S····Sb, formed a highly distorted octahedral geometry about the Sb while the two bridging interactions μ_2_—S····Sb in [SbCl(Me_2_DTC)_2_]n (1b) were distorted and also resulted in a square pyramidal geometry about the metal centre. This observed difference in intermolecular interactions establishes the two different polymorphs in their solid state. In the case of the bismuth complex {[Bi(Me_2_DTC)_3_]_2_}(2), the two bridging interaction μ_2_—S····Bi resulted in a pentagonal bipyramidal geometry around the bismuth metal. Similarly, complex {[Bi(Et_2_DTC)_3_]_2_}(3) is dimeric with two bridging μ_2_—Bi bonds of 3.188(1) Å, which resulted in a molecular conformation in the crystal structure. The geometry for each monomer unit has a six coordination about the Bi centre, with five sulphur atoms occupying the equitorial position while one sulphur atom is located in the axial position. The geometry about the Bi centre in complex {[Bi(Et_2_DTC)_3_]_2_}(3) was best described as pentagonal bipyramidal geometry [42].

**Figure 5:**
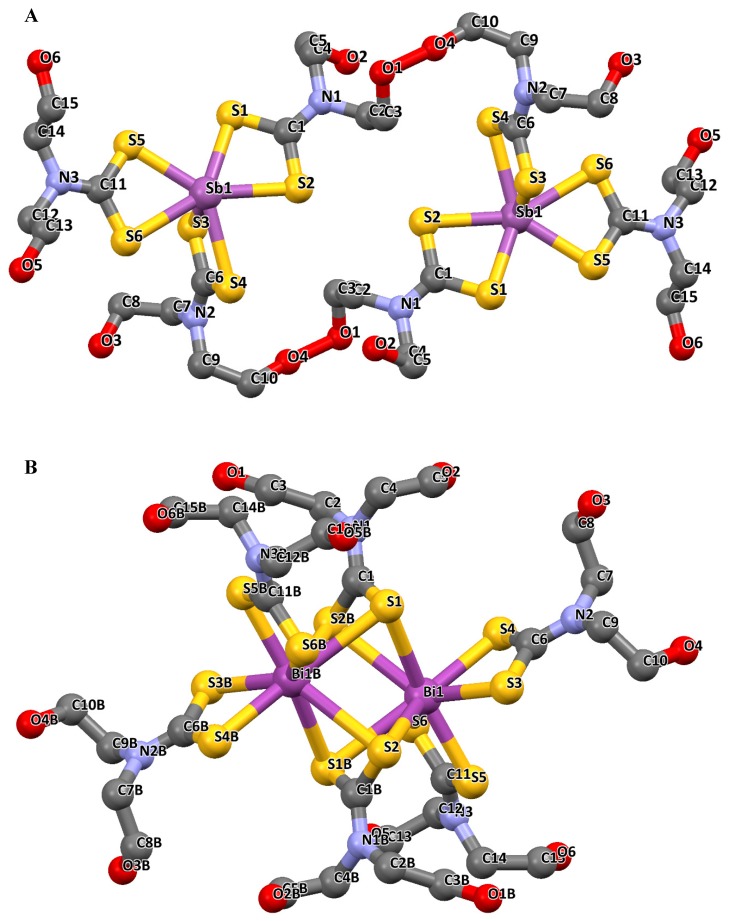
(**A**) Crystal structure and the labelling scheme of [Sb(deadtc)_3_]. (**B**) Crystal structure and the labelling scheme of the two crystallographyically independent units of [Bi(deadtc)_6_]. Unlabelled atoms are related by symmetry operation to labelled atoms. Redrawn from [60], with permission from Elsevier (copyright 2019).

Tris(*N*,*N*′-iminodiethanoldithiocarbamato) complexes of antimony and bismuth, represented as [Sb(deadtc)_3_] and [Bi(deadtc)_3_] respectively, have been reported [60]. In the coordination polyhedron of [Sb(deadtc)_3_], shown in Figure 5A, one of the Sb--S bonds was very short (2.46 A), and the differences between others were not significant due to their larger size. It was found that, where the length of two Sb---S bonds were similar, such as Sb--S(1) and Sb--S(2), the two C--S bonds were also almost equal; while in the situation where the Sb---S bonds were dissimilar, the differences between the Sb---S bonds were more pronounced such as for the Sb--S(5) and Sb--S(6) bonds. All the available sulphur atoms were bonded to the metal in a bidentate fashion which, therefore, confers coordination geometry about the antimony metal, best described as a distorted pentagonal pyramid. In the coordination polyhedron of [Bi(deadtc)_3_] shown in Figure 5B, two independent centrosymmetric molecules were found in the molecular structure of the complex so that the asymmetric unit is formed by one half of both molecules, resulting in four molecules per unit cell. According to the report, independent of the crystallographic views, all molecules were assumed to have comparable bond lengths and structure. All the available sulphur atoms were bonded to the metal in a bidentate fashion, with two of the six bidentate ligands bridging in such a way that each sulphur atom was simultaneously attached to both metal ions, and consequently conferred a distorted square antiprism geometry about the bismuth metal atoms [60]. In both complexes, the distortion from the expected ideal geometry observed was found to be due to the stereochemical active lone pair, which was very evident in the structure of the [Sb(deadtc)_3_] and [Bi(deadtc)_3_] complexes [1].

**Figure 6:**
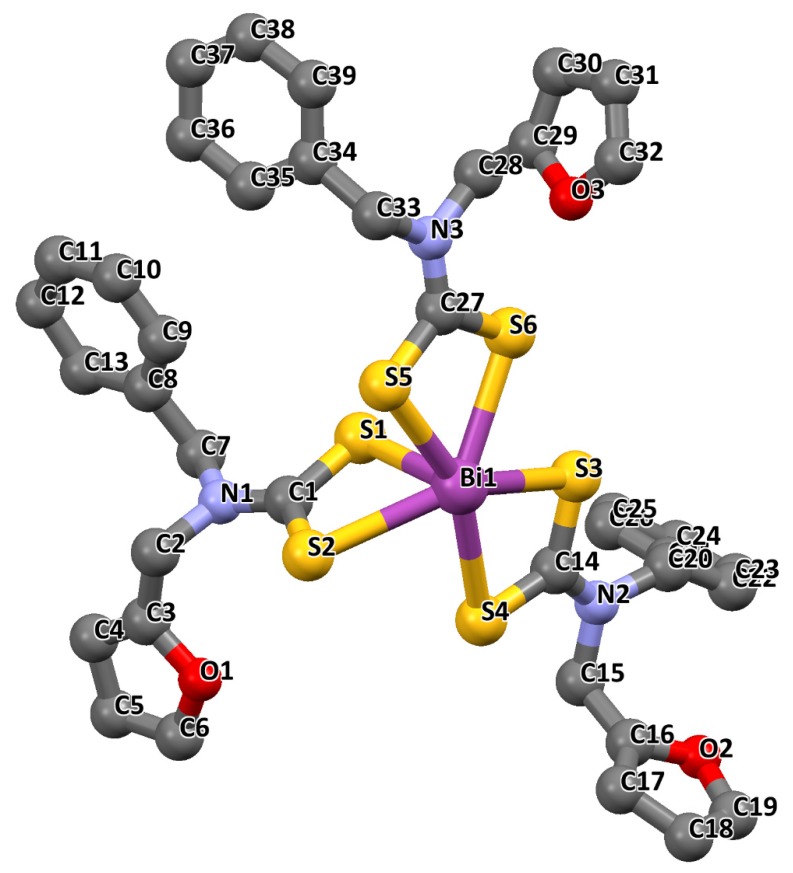
Crystal structure and the labelling scheme of tris(*N*-furfuryl-*N*-benzyldithiocarbamato-*S*,*S*′)bismuth(III) at 40% probability. Redrawn from [61], with permission from Elsevier (copyright 2019).

Tamilvanan et al. [61] reported the crystal structure of the tris(*N*-furfuryl-*N*-benzyldithiocarbamato-*S*,*S*′)bismuth(III) complex obtained in acetone. The structure of the complex, presented in Figure 6, showed that the Bi metal was coordinated in a bidentate fashion to six independent sulphur atoms. The Bi–S lengths were in the range 2.5876 (12) to 2.9055 (13) Å, with the shortest length Bi–S1 (2.5876 (12) Å) at the apex position. The other five sulphur atoms, in the equatorial plane, are, however, not coplanar and somewhat deviates from the expected 90°. This resulted in a distorted pentagonal pyramidal configuration about the Bi(III) atom via long range intermolecular Bi···S interactions [61].

**Figure 7:**
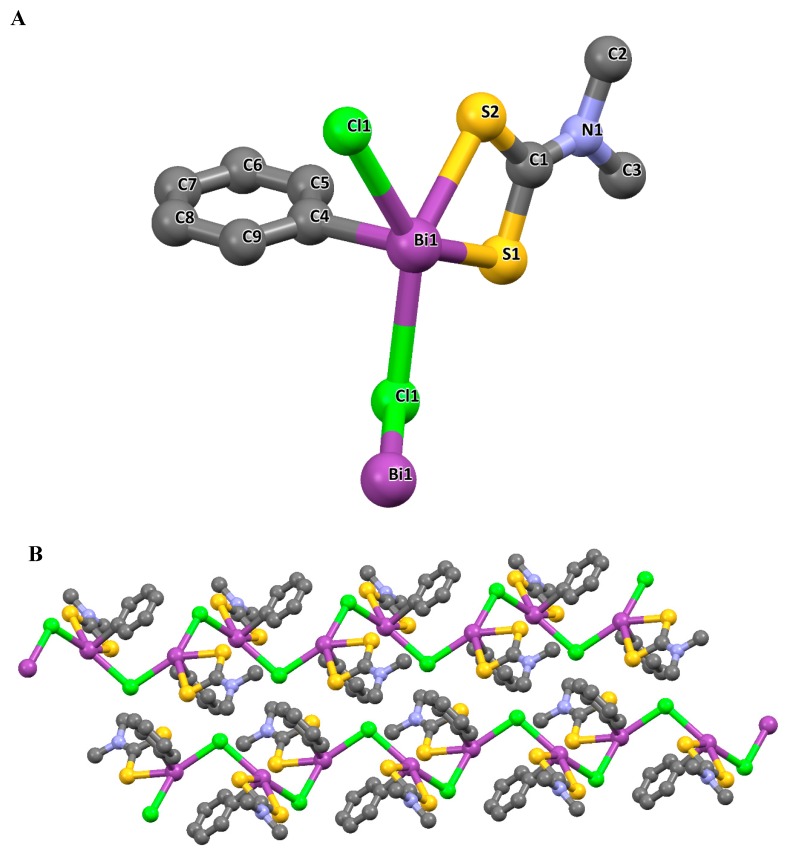
(**A**) Crystal structure of the complex and the labelling scheme [PhBiS_2_CN(CH_3_)_2_Cl]n. All hydrogen atoms are omitted for clarity. (**B**) 2D structure of the complex [PhBiS_2_CN(CH_3_)_2_Cl]n. All hydrogen atoms are omitted for clarity. Redrawn from [62], with permission from Taylor and Francis (copyright 2019).

The structures of some organometallic complexes of both Sb and Bi dithiocarbamate have been reported. The dithiocarbamate complexes of organobismuth with the oxidation states of +3 and +5 are quite rare. The few reported organobismuth complexes are in the +5 oxidation state. For example, Cui et al. reported a monophenylbimuth(V) polymer ([PhBiS_2_CN(CH_3_)_2_Cl]n) derived from a dithiocarbamate ligand of dimethyldithiocarbamate dihydrate and triphenylbismuth dichloride. The single crystal obtained showed the complex to be monoclinic, with a P2(1)/n space group (Figure 7). In this complex, each of the Bi ion was coordinated by five atoms; two sulphur atoms in a bidentate fashion, a carbon from the phenyl group attached to the Bi metal, and two chlorine atoms. The C4 atom of the phenyl group attached to the Bi centre was located in the apical position of the pyramid configuration, while all other atoms attached to the Bi were found at the base of the pyramid. All bond angles contributing to the observed configuration were not ideal and deviated from the expected 90°, which confers a distorted pyramidal geometry on the complex. Furthermore, a 1D spiral chain structure was formed due to the bridging chlorine atom linking the Bi metals, and two adjacent 1D spiral chain resulted in a form of a 2D network double helix structure [62].

**Figure 8:**
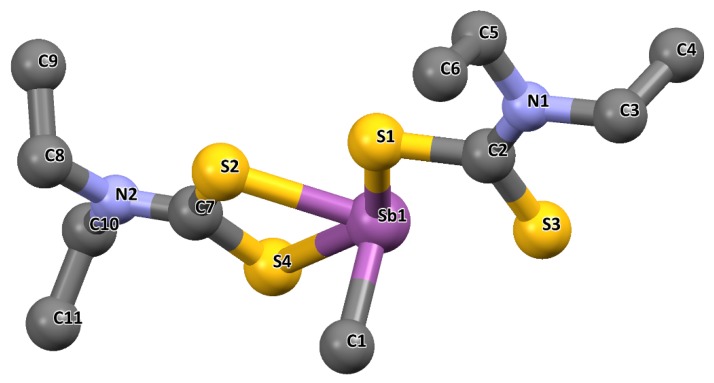
Crystal structure of the complex and the labelling scheme [CH_3_Sb(SaCNEt_2_)_2_]. All hydrogen atoms are omitted for clarity. Redrawn from [63], with permission from John Wiley and Sons (copyright 2019).

Similarly, the crystal structure of methylantimony(III) diethyldithiocarbamate, [CH_3_Sb(S_2_CNEt_2_)_2_] was reported by Weiber et al. [63]. This complex is monoclinic with four molecules per unit cell, and belongs to the space group P21/c (No. 14) (Figure 8). The dithiocarbamate ligands were asymmetrically chelated to the Sb atom. The fragments S4S2C7NC8C0 and S1S3C2N2C5C3 were within good approximation and enclosed an angle of 19.10° with each other. The carbon of the methyl group attached to Sb was approximately perpendicular to the plane of the four sulphur atoms with a deviation of 0.052 (1) A. No special intermolecular interactions occurred. The geometry about the central Sb also suggested a distorted pyramidal structure similar to others reported in the literature [62].

Diverse bonding modes and structural patterns occur in these groups of complexes, but a common observation is that both Bi and Sb complexes are often associated with interactions that lead to supramolecular assemblies [1]. Due to their different configurations and structures, which influence their electronic, physical, and chemical properties, they have found relevance in numerous biological applications and material syntheses, especially in their +3, as a single source precursor to metal sulphide nanoparticles of the type M_2_S_3_ [64].

## 3. Some Biological Potential of Bismuth and Antimony Dithiocarbamate Complexes

Metal containing drugs have continued to offer alternative therapies and advantages to the “conventional” organic drugs [65]. Generally, metal ions play important roles in biological systems. Their scarcity in the human body could result in diseases such as anaemia, growth retardation, heart diseases, and so on [66]. Therefore, it is expedient to recognize and understand, at least on a molecular level, their role in the body physiology of humans. The use of metal based drugs dates back to ancient times and has a rich and varied history [20]. As far back as 3500 BC, the Chinese and those in the Middle East made use of metal based drugs derived from gold and mercury chloride as a diuretic [20]. In the last century, specifically the early 1900s, Paul Elhrich used an arsenium based drug (arsenophenylglycine and arshphenamine) to treat trypanosoma disease (sleeping sickness) and syphilis, respectively [67]. This discovery birthed the development of ‘metallodrugs’ in that era, leading to more discoveries such as the use of sodium vanadate and derivatives of bismalltolato oxovanadium(IV) complexes for diabetics, and the use of gold based complexes such as aurothiomalate, auranofin, and aurothioglucose for rheumatoid arthritis [67]. Despite this early potential demonstrated by these metal based drugs, they were continuously overlooked in favour of organic based drugs until the serendipitous discovery of a platinum based anticancer complex, called cisplatin, in 1969. This discovery drastically increased the study and the use of metal based therapy to date, and in turn has resulted in the development of medicinal inorganic chemistry as a mature research discipline [67]. The idea behind the design of metal based drugs is very commendable. The careful selection of ligands (with desirable properties in geometries, coordination number, and redox state) and appropriate metals can lead to the regulation of the electronic, photophysical, and chemical properties in the body [20]. One might be tempted to overlook the impact of the ligands; however, they contribute greatly to the structural diversity, ligand exchange kinetics, modulate stability, and the second coordination sphere interactions [20].

Many approaches have been used in the synthesis and the application of metal based drugs and one of the most frequently used approaches originates from the idea of metal–organic drug synergism, which gives rise to two main effects: (i) the enhancement of the biological activity of the organic part of the drug due to the presence of a metal ion, which may increase the residence time and thus allow the drug to reach its biological targets; and (ii) modulation of the toxicity often associated with metal ions due to the presence of the organic drug moiety, which allows the metal ion to get to the targeted sites and makes it less available for side and other damaging reactions [14,67,68]. A variety of metal complexes derived from different groups in the Periodic Table, especially the transition metals, have been extensively studied for biomedical applications [69]. These biological applications are prompted by their activities as anticancer [70], antimicrobial [71], anti-inflammatory [72], anti-tuberculosis [73], antimalarial [74], antidiabetic [72], and antileishmanial [75] agents. Several bismuth and antimony complexes of different ligands have been explored for different biomedical applications.

**Figure 9:**
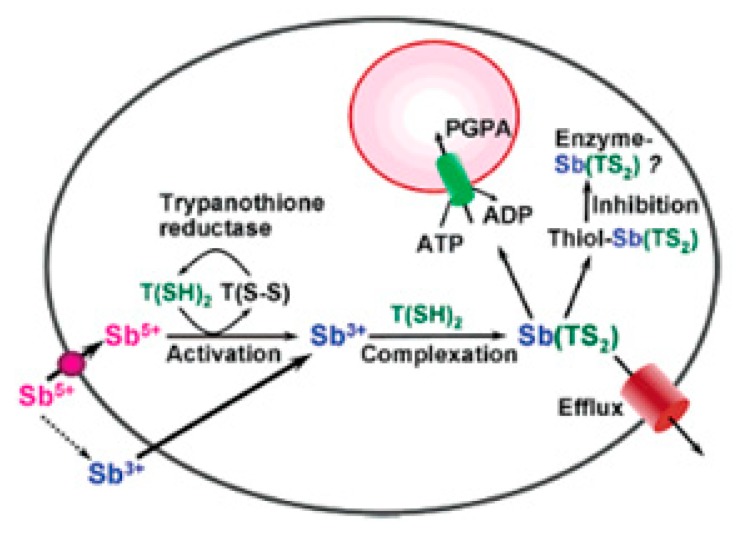
Proposed mechanism of action by various derivatives of antimony based compounds against leishmanial uptake into parasite. Reprinted (adapted) with permission from [80] American Chemical Society (copyright 2019).

Neither bismuth nor antimony is known to play any natural biological function. However, bismuth can be tolerated in large concentrations in the body, while antimony has a high toxicity comparable to arsenic [22]. Complexes of Sb and Bi have been observed to be clinically useful in the treatment of diverse infectious diseases [76]. Bismuth compounds have been continuously used as medicinal agents for over two centuries to treat medical disorders and, in recent times, some of these bismuth based drugs have now been commercialised such as DeNol and Peptobismol [22]. Several compounds containing bismuth complexes have shown promising in vitro activities against gastric ulcers caused by *Helicobacter pylori* as well as its usage as a radio-therapeutic agent for cancer treatment [26,76]. Similarly, there have been increased interest in the biological application of antimony based compounds and have been used against microbes and parasites [6]. Its major use, however, is in the treatment of leishmaniasis [31]. The antimony based drugs currently used for treatment of leishmaniasis include Glucantime (meglumine antimoniate) and Pentostam (sodium stibogluconate) [77]. The downside in the usage of these drugs is that the molecular mechanisms are still not completely clear and their activities are often accompanied with severe side effects related to toxicity [77]. Although the mechanism remains unclear, a hypothesis (see Figure 9) on the action of these antimony compounds, which is believed to follow a reduction or an activation process of Sb^5+^ to Sb^3+^ either enzymatically or non-enzymatically [78], has been proposed. The reduced Sb^3+^ then interferes with the trypanothione metabolism in the leishmania parasites, forming a complex with either glutathione or trypanothione. The reduced Sb^3+^ may sometimes form a ternary complex ((GS)Sb(TS)_2_) with monothiol ligands, GSH and T(SH)_2_. This complex (simple or ternary) can then inhibit enzymes such as trypanothione reeducates. In addition, the reduced Sb^3+^ may simultaneously be extruded by the As pump [78]. Similarities in the properties of antimony and bismuth has led to some studies aimed at evaluating the antileishmanial properties of Bi compounds [26]. For instance, some complexes of Bi have exhibited significant antileishmanial activity against the promastigotes of *L. major* V121 [7]. Hence, the bismuth compound could circumvent the toxicity associated with antimony compounds. Although many studies have been carried out on the evaluation of the antimony and bismuth complexes for antileishmanial activity, as at the time of this review, none of their dithiocarbamate complexes (both inorganic and organometallic) have been studied yet for these activities. Thus, this opens up an interesting area of research for the future, since dithiocarbamate compounds have already been established to show potentials such as antileishmanial agents [79].

Several bismuth based compounds have been found to be effective due to their in vitro antifungal, antiviral, and anticancer properties [9,76,80]. They have also been found to be useful, together with antibiotics, in the treatment of gastrointestinal diseases through the eradication of *Helicobacter pylori* [80]. Most Bi-based drugs used for the treatment of gastrointestinal diseases have also shown a complicated and unclear mechanism. They are generally believed to be taken up into gastric mucus to form a protective coating, probably as BiOCl and bismuth citrate complexes on the ulcer crater [80]. They may bind strongly to the proteins of the connective tissues, mucus glycoproteins, and enzymes and could inhibit *Helicobacter pylori* adherence [80]. From accumulative studies, proteins (peptides) have been found to be the potential targets of Bi based drugs. Different Bi based compounds have been shown to interact with a wide range of proteins such as metallothionein [65], lactoferrin [20], serum albumin [78], and human serum transferrin [20,78]. Since *H. pylori* utilises a specific lactoferrin for iron acquisition, the binding of Bi to this protein can deprive the acquisition of iron into its cell [20,78]. These drugs have also been found to inhibit several enzymes such as yeast alcohol dehydrogenase by interfering with the zinc site and ultimately altering the enzyme native structures [78].

Different biocidal activities have been reported for the trivalent Bi and Sb dithiocarbamate complexes. Although not many reports can be found on the antimicrobial studies of these complexes, few reports made on their activities have shown that these complexes have great potential as antimicrobial agents. Ariza-Roldán et al. [81] reported the synthesis and antimicrobial evaluation of some metal ephedrinedithiocarbamate complexes including both Sb(III) and Bi(III) derivatives. These complexes were screened against different Gram-negative (*Pseudomona aeruginosa*, *Escherichia coli*, *Klebsiella pneumoniae*, *Salmonella dublin*, and *Enterobacter cloacae*) and Gram-positive bacteria (different strains of *Staphylococcus aureus*, *Enterococcus caseofluvialis*, and *Staphylococcus sciuri*). In this study, all the complexes exhibited good activity against the screened bacterial strains. In general, between Bi and Sb complexes, the Sb(III) derivative showed better activity than its Bi(III) counterpart for the positive bacterial strains, while no appreciable activity was observed for the Bi(III) complex with the negative strains [81]. Furthermore, some monophenylantimony(III) compounds of cyclic dithiocarbamates have been prepared in 1:1 and 1:2 molar ratios, and screened for antimicrobial activities against *E. coli*, *P. aeruginosa* (Bacteria), and *A. niger* and *A. flavus* (Fungi) [51]. The obtained result showed that organometallic complexes were more inhibitory than their corresponding ligand based on Tweedy’s chelation theory; chelation reduces the polarity of the metal atom mainly because of the partial sharing of its positive charge with the donor group and possible electron delocalisation over the entire ring [51]. Consequently, the lipophilic character of the complexes is increased, which in turn, favours the permeability of the complexes over the layer of cell membranes. The final outcome also showed that the complexes still bearing the chloride group had a lesser activity than those without the chloride group.

Dithiocarbamate compounds have, in general, been found to exhibit higher cytotoxicity activity against cancerous cells than other corresponding sulphur based ligands like thiones (which is up to 1000-fold higher) [82]. In the use of Pt-based drugs as chemotherapeutics, compounds bearing sulphur donor groups have been used as chemo-protectants because of their ability to modulate nephrotoxicity [14]. Thus, dithiocarbamate complexes have great potential as cytotoxic agents against diverse cancerous cell lines. Ozturk et al. [42] carried out an in vitro cytotoxicity screening against human breast (MCF-7) and human cervix adenocarcinoma (HeLa) cells using both Sb(III) and Bi(III) dithiocarbamate complexes. These complexes showed higher activity than the standard anticancer agents such as cisplatin, doxorubicin, and tamoxifen. The complexes exhibited a 21–53-fold higher activity than cisplatin against HeLa cells, while against MCF-7 cells, it showed 158–340-fold higher activity. Antimony(III) and bismuth(III) complexes exhibited a stronger anti-proliferative activity against MCF-7 than against HeLa cells. Urgut et al. [82] reported three forms of Sb(III) dimethyldithiocarbamate complexes with good cytotoxicity; exhibiting 62–162-fold higher activity than cisplatin when screened against MCF-7 cells. Increased sensitivity was also observed towards HeLa cells than those against MCF-7. It was found that complexes with higher activity towards (MCF-7) cells exhibited low H-all intermolecular atoms interactions and the halogen ions present in the final molecule of the complexes, play no significant role in the cytotoxicity of the complexes [82]. Sb(III) S-benzyldithiocarbazate was synthesised and screened against some cancerous leukemic cells by Tarafder et al. [83]. These complexes showed some chemotherapeutic significance with CD_50_ values of 3.2 and 6.7 μg/mL, which are considerably lower than the free SbCl_3_, but higher than the S-benzyldithiocarbazate ligand. These enhanced activities have been attributed to the role of the nitrogen and sulphur nucleophilic sites in the complexes. Some Bi(III) complexes from different dithiocarbamate ligands (*N*,*N*-dialkyldithiocarbamates and *N*-alkyl-*N*-phenyldithiocarbamates) were synthesised and screened against an array of human cell lines including A498, renal cancer; MCF-7, oestrogen receptor (ER)+/progesterone receptor (PgR)+ breast cancer; EVSA-T, oestrogen receptor (ER)-/progesterone receptor (PgR)—breast cancer; H226, non-small cell lung cancer; IGROV, ovarian cancer; M19 MEL, melanoma; and WIDR, colon cancer by Li et al. [84]. A conspicuous observation from the screening results indicated that most of the synthesised complexes were more potent than the standard cisplatin drug, and also greater in activity than the standard organic drugs used in the study (doxorubicin, fluorouracil, and etoposide). Although some of the complexes showed no cytotoxic activities against the used cell lines, but some, on the other hand, showed good activities comparable in action to methotrexate. The in vitro cytotoxicity shown by this group of bismuth dithiocarbamate complexes is translated into anti-tumour activity such as the promising antitumoral activity observed in the trial involving [Bi(S_2_CNEt2)_3_] and the cisplatin-resistant ovarian cancer cell line, OVCAR-3 [84].

## 4. Conclusions

The salient features on the chemistry and the biological application of Bi(III) and Sb(III) dithiocarbamate complexes have been successfully highlighted in this review. Unlike their oxo-ligand derivatives, the dithio-derivatives were found to prefer discrete monomeric structures and, if there is any, a weak secondary interaction that inhibits the formation of a large molecule in a true sense. This has led to a wide variety of structural geometry due to their diverse bonding modes and structural patterns. A common phenomenon found in the complexes of both Bi and Sb is their ability to interact, which often leads to supramolecular assemblies. Hence, the coordination environment preferred by these metal atoms within these complexes have proven useful in the medicinal and biological context, showing a wide variety of biological properties such as antitumoral, antimicrobial, cytotoxicity, antileishmanial, and antiviral activities. Although, bismuth can be tolerated to a large amount in the body, antimony has high toxicity comparable to arsenic. Their complexes have, however, been found to be clinically useful in the treatment of several infectious diseases because of the synergistic action of modulating associated toxicity of the metal and the enhancement of the useful biological properties of the used organic ligands. Thus, as we continue to search for compounds with outstanding biological potentials, there is a need for the continuous synthesis of these complexes because only a few reports are available on both their synthesis and biological relevance in their +3 state and, even worse, their +5 state.
